# Point‐of‐care detection of fibrosis in liver transplant surgery using near‐infrared spectroscopy and machine learning

**DOI:** 10.1002/hsr2.1652

**Published:** 2023-10-31

**Authors:** Varun J. Sharma, John A. Adegoke, Michael Fasulakis, Alexander Green, Su K. Goh, Xiuwen Peng, Yifan Liu, Louise Jackett, Angela Vago, Eric K. W. Poon, Graham Starkey, Sarina Moshfegh, Ankita Muthya, Rohit D'Costa, Fiona James, Claire L. Gordon, Robert Jones, Isaac O. Afara, Bayden R. Wood, Jaishankar Raman

**Affiliations:** ^1^ Department of Surgery, Melbourne Medical School University of Melbourne Melbourne Victoria Australia; ^2^ Brian F. Buxton Department of Cardiac and Thoracic Aortic Surgery Austin Hospital Melbourne Victoria Australia; ^3^ Centre for Biospectroscopy Monash University Melbourne Victoria Australia; ^4^ Department of Engineering University of Melbourne Melbourne Victoria Australia; ^5^ Liver & Intestinal Transplant Unit Austin Health Melbourne Victoria Australia; ^6^ Department of Anatomical Pathology Austin Health Melbourne Victoria Australia; ^7^ Department of Microbiology and Immunology, Peter Doherty Institute for Infection and Immunity University of Melbourne Melbourne Victoria Australia; ^8^ DonateLife Victoria Carlton Victoria Australia; ^9^ Department of Intensive Care Medicine Melbourne Health Melbourne Victoria Australia; ^10^ Department of Infectious Diseases Austin Health Melbourne Victoria Australia; ^11^ School of Information Technology and Electrical Engineering Faculty of Engineering, Architecture, and Information Technology Brisbane Queensland Australia; ^12^ Biomedical Spectroscopy Laboratory, Department of Applied Physics University of Eastern Finland Kuopio Finland

**Keywords:** chemometrics, liver, near‐infrared spectroscopy, spectromics, transplant, vibrational spectroscopy

## Abstract

**Introduction:**

Visual assessment and imaging of the donor liver are inaccurate in predicting fibrosis and remain surrogates for histopathology. We demonstrate that 3‐s scans using a handheld near‐infrared‐spectroscopy (NIRS) instrument can identify and quantify fibrosis in fresh human liver samples.

**Methods:**

We undertook NIRS scans on 107 samples from 27 patients, 88 from 23 patients with liver disease, and 19 from four organ donors.

**Results:**

Liver disease patients had a median immature fibrosis of 40% (interquartile range [IQR] 20–60) and mature fibrosis of 30% (10%–50%) on histopathology. The organ donor livers had a median fibrosis (both mature and immature) of 10% (IQR 5%–15%). Using machine learning, this study detected presence of cirrhosis and METAVIR grade of fibrosis with a classification accuracy of 96.3% and 97.2%, precision of 96.3% and 97.0%, recall of 96.3% and 97.2%, specificity of 95.4% and 98.0% and area under receiver operator curve of 0.977 and 0.999, respectively. Using partial‐least square regression machine learning, this study predicted the percentage of both immature (*R* ^2^ = 0.842) and mature (*R* ^2^ = 0.837) with a low margin of error (root mean square of error of 9.76% and 7.96%, respectively).

**Conclusion:**

This study demonstrates that a point‐of‐care NIRS instrument can accurately detect, quantify and classify liver fibrosis using machine learning.

## 1 BACKGROUND

Liver disease is the 11th leading cause of mortality globally, with two million deaths annually and an incidence that has risen by over 500% in the last four decades.^1^ An aging society combined with rising rates of obesity, diabetes, and nonalcoholic steatohepatitis has resulted in liver transplantation being the second most common form of solid organ transplantation. However, less than 10% of global transplantation needs are currently met.

Many donor livers are currently declined on measures such as subjective visual assessment, or moderately sensitive imaging methods such as ultrasound, FibroScan^2^, and computed tomography which may only demonstrate positive findings with gross deformity.^3^, ^4^ These methods have marginal sensitivity and do not quantify the degree of fibrosis or steatosis,^5^ subject to interobserver bias and remain surrogates for tissue biopsies, the routine clinical use of which is hindered by traumatic tissue resection, time‐intensive histopathology, laboratory‐based processing and need for expert analysis by a histologist or pathologist with optical microscopy.^6^, ^7^ The nonspecific and delayed assessment of liver fibrosis and steatosis, amplified by rapidly increasing global disease burden, leads to an economic, social, and healthcare burden mounting into millions of dollars.^8^, ^9^ A point‐of‐care instrument for assessment of fibrosis and steatosis therefore has potential to increase the number of livers available for transplantation and bolster surgical outcomes.

Recent advancements in vibrational spectroscopy combined with machine learning make point‐of‐care real‐time diagnosis of liver fibrosis and steatosis possible.^10^, ^11^, ^12^, ^13^, ^14^, ^15^, ^16^, ^17^, ^18^ Historically spectrometric techniques, such as mass spectrometry, have been confined to laboratory use as they require extensive tissue preparation before analysis, and are time, resource, and personnel‐intensive.^19^ In contrast, vibrational spectroscopy techniques possess the potential of point‐of‐care applications. NIRS obtains absorption patterns from the delivery of laser or light waves, providing nonperturbative, rapid, and label free assessment of tissue structure and composition^15^, ^17^, ^20^, ^21^ at a molecular level.^22^, ^23^ There are emerging data demonstrating that it can discriminate fibrosis^11^ by exploring collagen subtypes,^24^, ^25^, ^26^, ^27^ cross linking,^28^, ^29^ and distribution.^27^, ^30^ Early investigations have shown promising results in identifying hepatic fibrosis^10^, ^14^, ^15^, ^16^, ^31^, ^32^, ^33^, ^34^, ^35^, ^36^ using machine learning techniques such as Stochastic Gradient Descent, Neural Networks, and Logistic regression. Spectroscopic studies in formalin‐fixed tissue^32^, ^33^ have demonstrated spectroscopy can distinguish fibrotic regions from neighboring hepatocytes. In frozen tissue,^34^, ^35^, ^36^ studies have been able to distinguish hepatocellular carcinoma from surrounding cirrhosis using a combination of Raman and Infrared spectroscopy. However, there is a paucity of data in analyzing fresh tissue, which poses a significant hurdle to clinical translation. We believe this can be overcome with NIRS as it doesn't require sample preparation. Due to greater penetration depth, NIRS lends itself to miniaturization since it is more resistant to losses in spatial resolution, and is comparatively low cost compared to spectroscopy using Mid‐IR (InfraRed) and Raman techniques.^37^

## 2 AIM

This study demonstrates, as a proof of concept, that NIRS scans using handheld instruments correlate with those of histopathology. This is the next iterative step to making real‐time diagnosis of fibrosis and steatosis in liver surgery possible.

## 3 EXPERIMENTAL PROCEDURES

### 3.1 Sample retrieval

**Figure 1:**
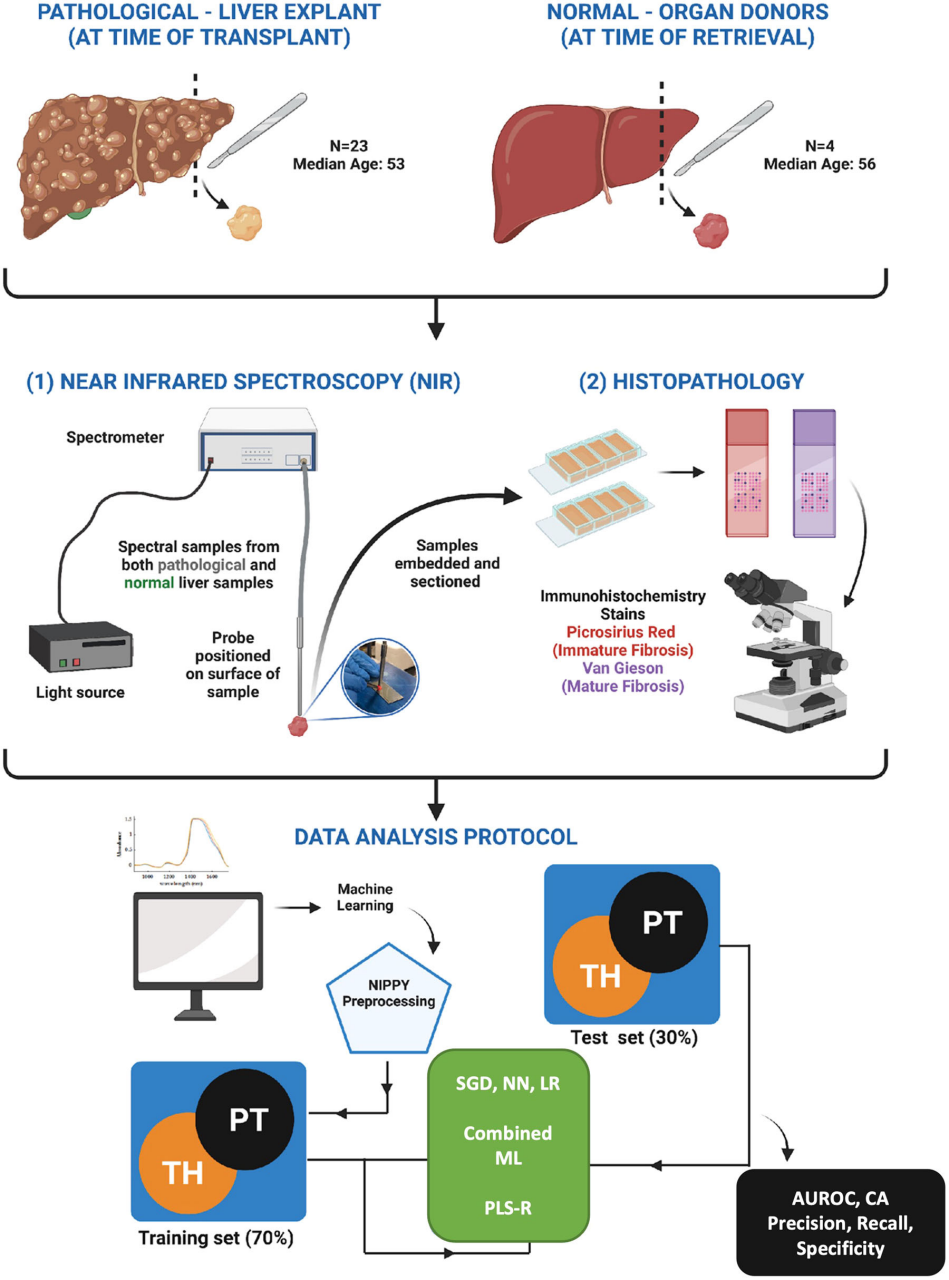
The method for tissue analysis. Samples were collected from both explanted livers at time of transplantation and control organ donor livers not suitable for transplant. All samples were analyzed using near infrared spectroscopy and then with histopathology with Picrosirius Red and Van Geison stains. Data from both were compared using artificial intelligence. Data were filtered using NIPPY preprocessing and split into training (70%) and test (30%) datasets. The model was assessed stochastic gradient descent (SGD), neural network (NN), logistic regression (LR), partial least square regression (PLS‐R) and a combined “combined ML” algorithm. The model was assessed using area under receiver operator curve (AUROC), classification accuracy (CA), precision, recall, and specificity.

An outline of the methods has been provided in Figure 1. Samples of 1 cm^3^ size from random positions within the liver (both surface and core) were obtained from both pathological and control patients. Pathological samples of liver were retrieved from the Victorian Liver Biobank of the Liver Transplant Unit (LTU), Austin Health, Melbourne, Victoria. LTU is the sole liver transplant center for the states of Victoria and Tasmania, Australia and prospectively stores samples from the time of liver explant as part of an ethically approved Liver Biobank. A series of 23 patients with liver disease were selected at random for use in this study.

Physiological control samples of liver were prospectively obtained from consecutive deceased organ donors by the Australian Donation and Transplantation Biobank (ADTB).^38^ These were acquired intra‐operatively at the time of organ retrieval in organ donors with consent for donation to the ADTB. Samples were transported on ice and immediately stored into 2 mL Eppendorf tubes, snap‐frozen in liquid nitrogen and stored in cryogenic tanks at −80°C.

For analysis, samples were thawed under standard laboratory conditions. All samples were deidentified and source blinded for all subsequent analysis.

#### 3.1.1 Demographics

In total, this study used a series of 27 patients, from which 107 samples were obtained. 100 of these were pathological samples were from 23 liver disease patients at the time of liver explanation and 20 fresh tissue samples from four organ donors whose liver did not meet viability criteria for transplantation at the time of DCD (donation after circulatory death) donation.

**Figure 2:**
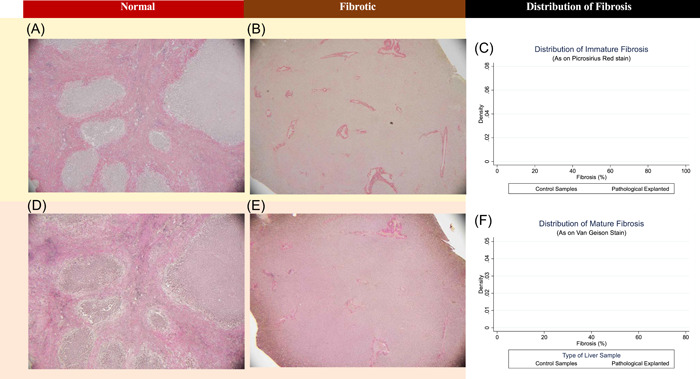
Distribution of quantified fibrosis in control donor and pathological explanted samples. Immature fibrosis stains, as seen on picrosirius red (PSR) stains are shown in (A) normal tissue, (B) fibrotic tissue with 60% fibrosis, with (C) distribution across of all samples in control (brown) and pathological (orange) samples. Mature fibrosis, as seen on Van Geison (VG) stains, are shown on (D) normal tissue, (E) fibrotic tissue with 60% fibrosis, with (F) distribution across of all samples in control (brown) and pathological (orange) samples.

The median age was 53 years (interquartile range [IQR] 40‐57) in liver disease patients and 56 years in organ donors, 66.7% (*n* = 18) male in liver disease cohort and 75% in the organ donor (*n* = 3) cohort. The indication for transplantation and other co‐morbidities are as described in Table 1. Of all explanted liver samples, 54% (*n* = 54) had cirrhosis, with a median quantified immature fibrosis of 40% (IQR 20–60) and mature fibrosis of 30% (10%–50%). Explanted samples represented multiple stages of fibrosis, with the percentage of fibrosis ranging from 0% to 90%, and exhibiting a normal distribution as shown in the kernel density plots (Figure 2A–F). The donor livers were noncirrhotic, with median fibrosis (both mature and immature) of 10% (IQR 5%–15%).

**Table 1:** Demographic details of patients from whom liver samples were acquired, stratified by pathological samples from end‐stage liver failure patients and normal controls from organ donors.

Variable	Pathological	Normal control	*p*‐Value
	(*n* = 23)	(*n* = 4)	
Demographics			
Age	53 (40–57)	56 (50–62)	0.41
Male sex	14 (60.8%)	3 (75.0%)	0.71
Diagnoses			
Acute liver failure	4 (17.4%)	0 (0.0%)	
Amyloidosis	1 (4.3%)	0 (0.0%)	
Cirrhosis	13 (56.5%)	0 (0.0%)	NA
Hepatic artery thrombosis	1 (4.3%)	0 (0.0%)	
Hepatocellular cancer	1 (4.3%)	0 (0.0%)	
Other/unknown	3 (13.0%)	0 (0.0%)	
Etiology			
Alcoholic liver disease	3 (13.0%)	0 (0.0%)	
Autoimmune hepatitis	2 (8.7%)	0 (0.0%)	
Familial transtherin amyloidosis	1 (4.3%)	0 (0.0%)	
Hepatitis A	1 (4.3%)	0 (0.0%)	
Hepatitis B	2 (8.7%)	0 (0.0%)	
Hepatitis C	7 (30.4%)	0 (0.0%)	NA
Wilson's disease	1 (4.3%)	0 (0.0%)	
Primary sclerosing cholangitis	1 (4.3%)	0 (0.0%)	
Primary biliary cirrhosis	1 (4.3%)	0 (0.0%)	
Nonalcoholic steatohepatitis	1 (8.7%)	0 (0.0%)	
Other/unknown	3 (13.0%)	0 (0.0%)	
Analysis			
Number of samples	100	20	NA
Number of spectra	565	120	
Presence of cirrhosis (>30% fibrosis)	54/100 (54.0%)	0/20 (0.0%)	<0.01
Median quantified immature fibrosis (IQR)	40% (20–60)	10% (5–15)	<0.01
Median quantified mature fibrosis (IQR)	30% (10–50)	10% (5–15)	<0.01

*Note*: The extent of fibrosis and cirrhosis from each sample was histopathologically validated.

### 3.2 Near‐infrared spectroscopy (NIRS) scans

Liver samples underwent a 3‐s scan using a handheld near‐infrared spectrometer (DWARF‐Star‐NIR, StellarNet incorporated, Carlson Circle, Tampa, Florida). This instrument was equipped with high performance InGaAs (Indium Gallium Arsenide) detectors and halogen light source (AvaLight HAL‐(S)‐mini, Avantes BV, Netherlands). A thermo‐electric cooling system was integrated with the detector to ensure stable temperature (±0.1°C) was maintained. Spectral data (wavelength range: 900–1700 nm, resolution ≈ 2 nm) were acquired via a custom‐built reusable stainless‐steel fiber optic probe (Avantes BV). Spectral acquisition was done by a software program written in Python (Python Software Foundation. Python Language Reference, version 3.1) and operated over a raspberry *Pi* Moodle.

#### 3.2.1 Histopathology (Gold Standard)

Results from NIRS Scans were compared to the gold standard of histopathology. Samples were embedded in paraffin, sectioned and stained with Haemotoxylin and Eosin, picrosirius red (PSR) and Van Geison's (VG) stains for assessment of fibrosis. Samples were assessed visually for fibrosis and steatosis by a blinded Anatomical Pathologist (Department of Anatomical Pathology, Austin Health, Melbourne, Victoria) to assess the presence of fibrosis (defined as >30% fibrosis), grade of fibrosis using the METAVIR (meta‐analysis of histopathology data for viral hepatitis) scale^39^, ^40^ (F0–F4), underlying diagnoses and percentage of both immature (PSR stain) and mature (VG stain) fibrosis using standardized international guidelines.^41^

#### 3.2.2 Clinical data analysis

Study data were collected and managed using Research Electronic Data Capture (REDCap) electronic data capture tools hosted at ADTB. Analysis of clinical data was done using Stata *v*15.0 (StataCorp. 2017. Stata Statistical Software: Release 15. College Station, TX: StataCorp LLC), with clinical variables reported as either counts with corresponding percentages, or median averages with IQR. Differences between sexes were assessed using student *t*‐tests for proportions and rank‐sum tests for continuous variables. Correlations between clinical and pathological variables were carried our using linear regression analysis and reported as correlation coefficients and 95% confidence intervals.

#### 3.2.3 Machine learning

First, NIR scans were visualized using PLS toolbox (Eigenvector Research Inc), an extensive suite of machine learning and statistical tools for advanced data analysis, which can be operated within the MATLAB environment (MathWorks). This study employed the following data cleaning strategies: (i) Data were visually examined to ensure that all absorption bands were consistent with those reported in literature.^25^, ^29^, ^32^, ^33^, ^34^ (ii) Principal component analysis (PCA) was employed for outliers' detection and the resulting hotelling's T‐squared distribution (T2) and leverage score were used to determine outliers. (iii) All outliers were excluded while the rest of the data set was passed on for modeling. Optimal pre‐processing steps to obtain smoothed spectra were determined via Nippy python module.^42^, ^43^

NIRS were entered into a standardized preprocessing algorithm using Quasar.^42^, ^43^ This was done by keeping spectra from 600 to 1750 nm, Savitzky‐Golay filter (window = 15, polynomial order = 2, derivative order = 2), area normalization peak from 0 and baseline correction. Data were then entered into Stochastic Gradient Descent (SGD), neural networks (NN), logistic regression (LR) machine learning (ML) algorithms using a 70–30 training test data split.^44^, ^45^ SGD used an optimal learning rate with 1000 iterations and a tolerance of 0.001, Lasso (L1) regularization with strength 0.01, and the Loss function was classified by Squared loss with Huber regression at 0.1. LR had regularization type Lasso (L1) and strength *C* = 3. SVM used v‐SVM with regression cost = 1.0, complexity bound = 0.5, RBF Kernel with g = auto, numerical tolerance of 0.01 and iteration limit of 100. The Combined ML algorithm combines all methods and is reported separately. Averages of the technical replicates acquired from each sample were used to avoid overfitting of models thereby avoiding the “technical replicate trap.” Model accuracy was assessed using precision, recall, specificity, and area‐under‐curve (AUC) of corresponding receiver‐operator‐curves (ROC). The diagnostic capability of the model was assessed using confusion matrices with classification rates. To assess the percentage of fibrosis, we used partial least square regression (PLS‐R). PLS‐R score plots of the model (latent variable = 6) were developed using the leave one out cross validation (LOOCV). The PLS‐R models were evaluated using the correlation coefficient (*R* ^2^), the root‐mean‐square error (RMSE).

## 4 RESULTS

### 4.1 Identifying Cirrhosis

**Figure 3:**
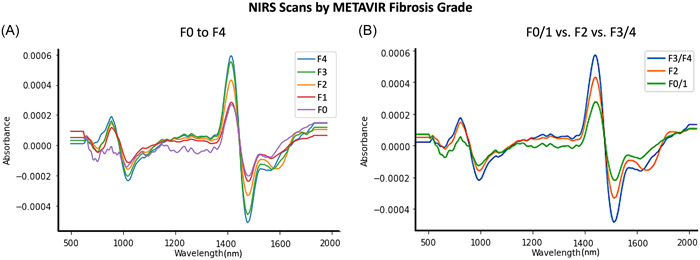
Averaged and postprocessed near infrared spectroscopy scans from (A) each METAVIR grade of fibrosis and (B) F0/F1 versus F2 versus F3/F4 METAVIR grades. AU, absorption unit; nm, nanometers.

This study undertook NIRS scans of all 107 samples, with representative spectra for each grade of fibrosis (F0–F4) after preprocessing shown in Figure 3. There was a notable difference in the NIRS absorption spectra between cirrhotic (F4) and disease‐free samples (F0). These are most prominent in regions typically associated with collagen (1484 and 1585 nm).^46^ This suggests that the most prominent distinguishing feature between NIRS scans of different fibrosis grades are regions associated with collagen.

### 4.2 Detecting fibrosis

**Figure 4:**
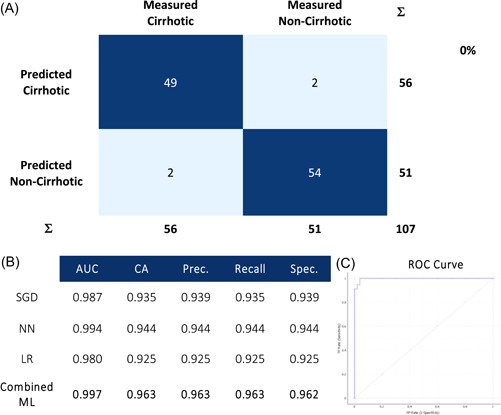
Near infrared spectroscopy scans ability to predict the histopathological presence of cirrhosis with (A) confusion matrices using a combined machine learning algorithm, (B) metrics of performance, and (C) corresponding receiver operator (ROC) curve. AUC, area under ROC curve; CA, classification accuracy; LR, logistic regression; NN, neural network; Prec., precision; SGD, stochastic gradient descent; Spec., specificity.

**Figure 5:**
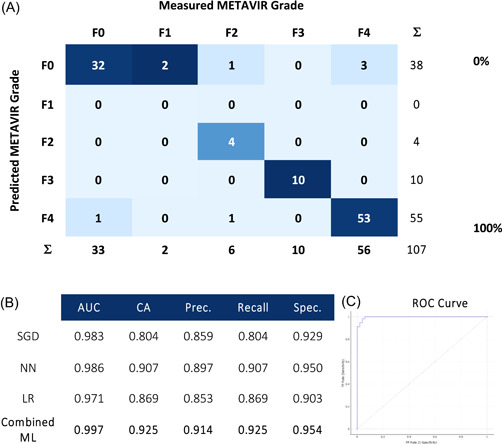
Near infrared spectroscopy scans ability to predict histopathological METAVIR grade of fibrosis, with (A) confusion matrices using a combined machine learning algorithm, (B) metrics of performance, and (C) corresponding receiver operator (ROC) curve. AUC, area under ROC curve; CA, classification accuracy; LR, logistic regression; NN, neural network; Prec., precision; SGD, stochastic gradient descent; Spec., specificity.

**Figure 6:**
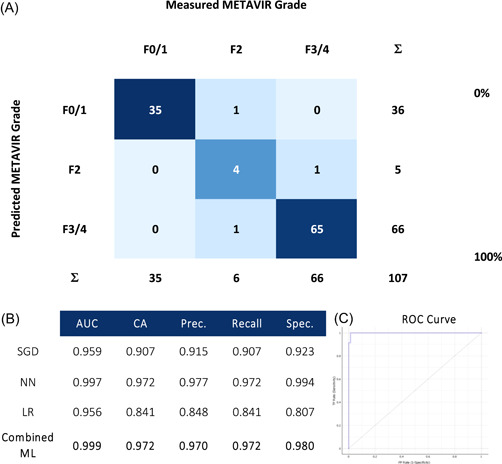
Near infrared spectroscopy scans ability to predict histopathological METAVIR grade of fibrosis, classified as F0/1 versus F2 versus F3/4, with (A) confusion matrices using a combined machine learning algorithm, (B) metrics of performance, and (C) corresponding receiver operator (ROC) curve. AUC, area under ROC curve; CA, classification accuracy; LR, logistic regression; NN, neural network; Prec., precision; SGD, stochastic gradient descent; Spec., specificity.

**Figure 7:**
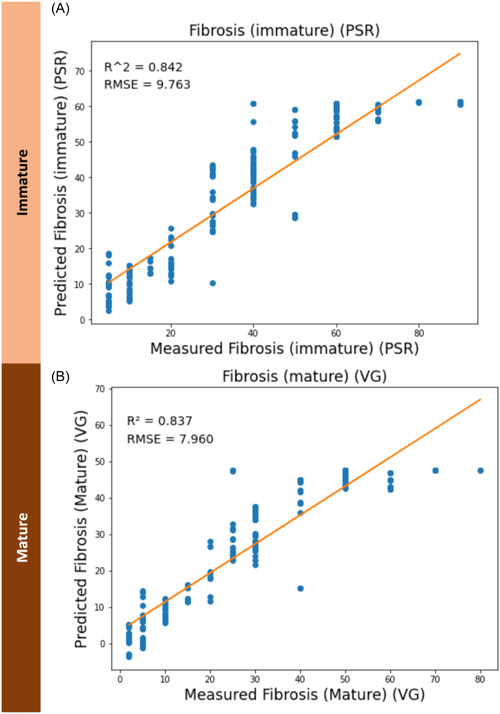
Partial least square regression analysis using near infrared spectroscopy data for predictions and histopathology for the ground truth. Predictions are shown separately for (A) immature fibrosis, as quantified on picrosirius red stains and (B) mature fibrosis, as quantified on Van Geison stains. PSR, picrosirius red; RMSE, root mean square error; VG, Van Geison stain.

Data from NIRS scans were entered into ML algorithms (Figures 4, 5, 6, 7) to assess its ability to identify the fibrosis. Confusion matrices compare ML predictions to measured values using histopathology and are presented with metrics of performance and corresponding receiver operator curves (ROC).

In distinguishing cirrhotic tissue, the ML models produced AUROC of 0.997, with a classification accuracy (CA), precision and recall of 96.3% and specificity of 96.2%. In identifying grades of METAVIR fibrosis, there was an AUC of 0.997, with classification accuracy of 92.5%, precision of 91.4%, recall of 92.5% and specificity of 95.4%.

Separate analyses were undertaken for identify tissue representing no or early (F0/1), intermediate (F2), and advanced fibrosis (F3/4) (Figure 6). This model produced an AUC of 0.999, with a classification accuracy of 97.2%, precision of 97.0%, recall of 97.2%, and specificity of 98.0%.

### 4.3 Quantifying fibrosis

We entered the blinded NIRS Scan data into a partial‐least square regression (PLS‐R) ML model to assess its ability to quantify fibrosis (Figure 7). In predicting immature fibrosis (PSR stain data), we obtained an *R* ^2^ of 0.842 in with a margin of error of 9.763% (RMSE). For mature fibrosis, we obtained an *R* ^2^ of 0.837 with a margin of error of 7.96% (RMSE).

## 5 DISCUSSION

We use a point‐of‐care NIRS instrument to scan fresh pathological and normal liver tissue from explanted livers, all of which are then compared to findings of conventional histopathology, with the following key findings. First, the NIRS spectra for diseased (F4 cirrhosis) and normal (F0) are unique at a crude visual level. Second, when we combine the spectral data with machine learning algorithms, NIRS has an accuracy of 96% in identifying cirrhosis and greater than 93% in grading or classifying the degree of fibrosis. Third, we find that we find that NIRS can accurately predict both immature and mature fibrosis (*R* ^2^ > 0.80) to within 10% (RMSE < 10%).

This study is the first to describe point‐of‐care NIRS in fresh human tissue to detect hepatic fibrosis. Current data for use of spectroscopy for analyzing fibrosis in biological tissue are emerging, but the majority use bulky benchtop instruments that are restricted to laboratory use.^10^, ^11^, ^12^, ^13^, ^14^, ^15^, ^16^, ^17^, ^18^ This is one of a small number of studies where miniaturized handheld instruments that carry point‐of‐care potential. However, most studies have used Raman spectroscopy, where we use NIR spectroscopy which benefits from greater penetration depth and reduced fluorescence of thermal perturbance of underlying tissue. Furthermore, the majority of previous studies were performed on animal tissue, fixed tissue or were based on less than 10 samples. In scanning 107 fresh samples with a 3‐s NIRS scan with a high degree of accuracy, we believe we are the first to demonstrate NIRS as a potential point‐of‐care clinical instrument for hepatic fibrosis, especially in transplant surgery.

The clinical implications for such a tool are considerable. At the time of retrieval, point‐of‐care NIRS could provide a rapid assessment of fibrosis, potentially predicting biopsy results, and therefore risk of adverse events; findings of viability may help to increase the number of livers available for transplantation (i.e., transplanting livers that would otherwise be rejected). The accuracy point‐of‐care NIRS exceeds other clinical assessments, such as Fibroscan which has a reported AUC of 0.70–0.89.^47^ Furthermore, our findings are from a heterogenous range of aetiologies, including hepatitis, alcoholic liver disease and nonalcoholic steatohepatitis; all of which have historically been difficult to diagnose using other imaging modalities. As samples have been obtained using a fibreoptic probe with a diameter less than 10 mm, with the technique being nonperturbative and autoclave safe, it can be safely applied to whole organs without the need for tissue excision. This has the potential for use with a minimally invasive laparoscopic approach.

Our study is unique due to the large number of fresh pathological and control human liver samples studied, which are typically challenging to obtain.^36^. NIR instrumentation is readily available, as it is already used extensively in industrial chemistry, with multiple combinations available for spectrometers, light sources, and fiber‐optic probes.^37^ Costs are under 20,000 USD, which are still cheaper than many of the imaging instruments used in clinical practice. Training to use instruments is becoming increasingly easier, with some instruments now available via smartphone applications with a “single‐click” user interface. The spectral range from 900 to 1700 nm and 785 scans, provides ample data for machine‐learning algorithms to yield high levels of accuracy. This allows us to not only diagnose fibrosis, but also quantify and typify it. With larger‐scale studies with in vivo validation, this is a tool that has immediate clinical applications. Future studies would also benefit from multi‐modal spectroscopic unsupervised evaluation and, that could compile a complementary array of data from Raman and Infrared (mid‐IR and near‐IR) spectroscopy. External validation at other centers with other instruments would also be required to validate the findings from this report.

## 6 CONCLUSION

This study demonstrates a point‐of‐care NIRS instrument can accurately detect, quantify, and classify liver fibrosis with the aid of machine learning algorithms.

## AUTHOR CONTRIBUTIONS

**Varun J. Sharma**: Conceptualization; data curation; formal analysis; funding acquisition; investigation; methodology; project administration; resources; software; supervision; validation; visualization; writing—original draft; writing—review and editing. **John A. Adegoke**: Data curation; formal analysis. **Michael Fasulakis**: Formal analysis. **Alexander Green**: Formal analysis. **Su K. Goh**: Data curation. **Xiuwen Peng**: Formal analysis. **Yifan Liu**: Formal analysis. **Louise Jackett**: Data curation; formal analysis. **Angela Vago**: Formal analysis; funding acquisition. **Eric K. W. Poon**: Formal analysis; funding acquisition. **Graham Starkey**: Formal analysis; writing—review and editing. **Sarina Moshfegh**: Data curation; formal analysis. **Ankita Muthya**: Data curation. **Rohit D'Costa**: Methodology; project administration; writing—review and editing. **Fiona James**: Data curation; funding acquisition; investigation; methodology; project administration; writing—review and editing. **Claire L. Gordon**: Data curation; formal analysis; funding acquisition; investigation; methodology; project administration; resources; supervision; visualization; writing—original draft; writing—review and editing. **Robert Jones**: Methodology; resources; supervision; visualization. **Isaac O. Afara**: Formal analysis. **Bayden R. Wood**: Formal analysis; supervision. **Jaishankar Raman**: Conceptualization; data curation; formal analysis; funding acquisition; investigation; methodology; project administration; resources; software; supervision; writing—original draft; writing—review and editing.

## CONFLICT OF INTEREST STATEMENT

The authors declare no conflict of interest.

## ETHICS STATEMENT

This study was approved by the Human Resources and Ethics Committee (HREC) at Austin Hospital, Heidelberg, Melbourne, Victoria (HREC/73660/Austin‐2021). Approval acquisition of human tissue from organ donors was as part of the Australian Donation and Transplantation Biobank (HREC/4814/Austin‐2019) and Donate Life Victoria (DLV) through the Australian Red Cross Lifeblood Health Human Research and Ethics Committee (Ethics 2019#08).

## TRANSPARENCY STATEMENT

The lead author Jaishankar Raman affirms that this manuscript is an honest, accurate, and transparent account of the study being reported; that no important aspects of the study have been omitted; and that any discrepancies from the study as planned (and, if relevant, registered) have been explained.

## Data Availability

The data that support the findings of this study are available from the corresponding author upon reasonable request.
